# Clinical Lectures

**Published:** 1861-01

**Authors:** Austin Flint

**Affiliations:** Prof. of Clinical Medicine and Medical Pathology, in the N. O. School of Medicine


*Clinical Lectures. Delivered at the N. O. Charity Hospital*

By Austin Flint, M. D., Prof. of Clinical Medicine and Medical Pathology, in the N. O. School of Medicine.

### LECTURE I.

### ON PULMONARY EMPHYSEMA.

*Embarrassed respiration and lividity not distinctive of Emphysema.—Mode of determining their true cause.—Existence of Pleurisy with effusion.—Anatomical characters of Emphsema.—Condition of the air-cells and tubes.—The condition of the lung explains the dyspnœa lividity.—History of a case.—Its Treatment.*

Gentlemen—In my last clinical lecture, I invited your attention to a patient in one of my wards who was suffering greatly from dyspnœa, and who presented considerable lividity of the prolabia and face. These were the most prominent of the symptoms in the case. The respiratory function was seriously compromised. The suffering in consequence was great, and the lividity showed incompleteness of the normal blood-changes which should be wrought by the interchange of certain elements with the atmosphere.

I stated to you that this patient was effected with pulmonary emphysema, and that the case would probably end fatally within a short time. This prognosis has been fulfilled. Death took place the day before yesterday. The dyspnœa increased, lividity became more marked, and the patient died from asphyxia and exhaustion combined. The contents of the chest have been removed, are on the table before me. I shall devote this lecture to some remarks on emphysema in connexion with the history of this case, and the morbid appearances presented after death.

The prominent symptoms in this case were embarrassed respiration and lividity. Now these symptoms are not distinctive of emphysema. We could not predicate on them the existence of emphysema. These symptoms are present in a variety of affections; they occur in certain diseases of the heart; in pneumonia when it extends over a large portion of the lungs; in acute phthisis; in pleurisy with large effusion; in capillary bronchitis, asthma, and obstruction of a bronchus. How did we arrive at the conclusion that the symptoms were not incident to any of these affections, but to emphysema? The history and associated symptoms enabled us to exclude readily some of them. The dyspnœa had existed continuously for too long a period to be consistent with the diagnosis of pneumonia, acute phthisis, capillary bronchitis, or asthma. Moreover, other symptoms which we should expect to find in connexion with either of these affections, were wanting. Thus, reasoning by way of exclusion, we could readily eliminate these; but the other affections named were not so readily excluded by the history and symptoms. It was necessary to call to our aid the results of a physical exploration of the chest. Having recourse to these, we were able to exclude without difficulty other of the affections. Disease of the heart, pleuritic effusion, and bronchial obstruction, were eliminated by finding certain signs wanting which should have been present, had these affections existed. In this way, we reached the conclusion that the prominent symptoms in the case were due to emphysema, employing a method of reasoning often extremely serviceable in the discriminaton of diseases. But the diagnosis was rendered more complete by the presence of certain physical signs which positively denoted the existence of emphysema, and the examination after death has verified the correctness of the diagnosis.

Let me now direct your attention to the anatomical characters of emphysema, as presented in the lungs before. But before speaking of these, I will refer you to certain appearances which show that at some period of the life of the patient, not very recent, he was affected with pleurisy. This is a fact of interest, since it is very probable that the attack of pleurisy may have had something to do with the production of the emphysema. In removing the lungs from the chest, they were strongly adherent to the thoracic walls on the left side, and entirely free from adhesion on the right side. You observe that the whole of the left lung is invested with a firm false membrane, which is attached to the visceral pleura by an adventitious structure resembling the areolar tissue.—

Over the lower part of the lung the false membrane is thick and leathery, and on slitting it up, several ounces of a puruloid liquid escape. This liquid is exterior to the lung, being situated between the false membrane and the pleura covering the lung. It is the residue of a liquid effusion which was probably large when the patient was affected with pleurisy, before the pleuritic adhesion had taken place. This liquid still separates the false membrane from the visceral plerua in some small spaces. Directing attention to the lungs, you perceive that they are notably voluminous. In a healthy chest, when it is opened, the lungs, if not adherent to the thoracic wall, collapse more or less, leaving a vacant space between them and the walls of the chest. The lung in this case was not collapsed on the right side, in which no adhesions existed. It is now two days since the organs were removed, and still, as you see, their volume is remarkably large. They look as if they had been artificially inflated.—This is one of the characters of emphysema, the special anatomical condition consisting in permanent dilatation of the air cells. The lungs were abnormally expanded during life, owing to the dilatation of the chest. They remain so after death. The elasticity belonging to these organs in health is so much impaired that the expanded volume continues, notwithstanding they have been handled considerably, and if suspended they would retain much of their present volume dried. Another of the characters belonging to lungs affected with emphysema is an exsanguine appearance. They appear to be absolutely devoid of blood. Even in the posterior portions, which are usually found to be congested after death, the vessels seem to be empty. This represents to some extent, a morbid state, existing, as we shall presently see, during life. Another character is a doughy or cushionly feel, which you will find to be well marked in this specimen. Another character is a remarkable dryness of the tissues; when a section is made no liquid escapes, and very little is squeezed out by pressure. These are the obvious characters of emphysema. On close examination of cut surfaces, in some specimens, the cells are seen to be enlarged, and sometimes cavities greater or less in size and more or less numerous, are apparent. These are caused by destruction of the cell walls and coalescence of the cells.—They are especially conspicuous in dried preparations. I have a beautiful specimen of this description which I obtained in this hospital last winter; the lungs presenting, on section, an appearance of these organs in the batrachian species of animals. Cavities of large size are occasionally produced by coalescence of the cells. These appearances are not presented in this specimen. The cells seem to be simply and uniformly dilated.

Emphysematous dilation may be limited or diffused over the whole lungs. In this specimen it extends over the whole of the right lung, but is relatively greater in the anterior and superior portion, as is usually the case. On the left side it extends over the whole upper two-thirds of the lung, and the lower third, instead of being expanded, is contracted.—On cutting into this contracted portion, the substance of the lung is found to be condensed. It is carnified, and contains, for the most part, no air. This appearance claims notice as perhaps having a bearing on the production of the emphysema. On making repeated sections into the carnified portion of the lung, I find an oblong, irregular cavity, which appears to be lined with a membrane, from which I scrape a bloody, mucous-like substance. This has not the appearance of a tuberculous cavity. It is probably a dilated bronchial tube. I find no tubercles present anywhere. The bronchial glands are enlarged. Here, at the bifurcation of the trachea, on each side, is an enlarged gland the size of an almond. On cutting into it, an abundance of carbonaceous matter is apparent.

Let us observe the condition of the air tubes. Following the trachea to the bronchi, and the sub-divisions of these as far as they can be traced, they present no appearance of contraction or obstruction. The mucous membrane everywhere is covered with a thick layer of mucous. When this is scraped away the membrane is intact. On the right side the membrane is reddened. The membrane seems to be thickened, but it is consistent, and there are no ulcerations.

The heart has been removed, attached to the lungs by its vessels. It is about the normal size. I open the cavities.—The walls are about the normal thickness, and present the appearance of healthy, muscular structure. All the cavities contain some loose, soft, black coagula, and each ventricle contains, also, a colorless clot entertwined with the tendinous cord, and from the left ventricle projecting for several inches into the aorta. These clots suggest remarks which I defer for some other occasion. All the valves of the heart are sound.

Reverting to the condition of the lungs, can we explain the rationale of the prominent symptoms in the case, viz. the dyspnœa and lividity? By reference to this condition, a little attention will, I think, render the connexion sufficiently intelligible. The effect of a permanent dilatation of the air cells is an abnormal expansion of the lungs during life, continuing, as we have seen, after death. Owing to the loss of elasticity, the lungs no longer collapse in the absence of a force producing their expansion. What is the effect upon the respiratory movements? The range of the expansion of the chest is diminished. The permanently expanded lung, in the first place, limits the expiratory movements; the chest contracts less with the acts of expiration, and, consequently, the inspiratory movements are restricted. The chest is habitually in the state of an inspiratory act partially performed; or, in other words, the expiratory act is habitually performed incompletely. You see, with a moment's reflection, that in this way the range of the alternate expansion and contraction of the chest in the alternate acts of inspiration and expiration, is diminished in proportion to the extent of the permanent expansion of the lungs. I can illustrate the point which I wish to explain by this pair of bellows. I separate the handles of the bellows as widely as possible, and thereby draw in a certain quantity of air; then I bring the handles as near together as possible, and I expel the air from the bellows. It is true that the comparison is not exact, because the chest is never so completely voided of air as the bellows, but the parallel is sufficient for the illustration. Now, suppose there is some obstacle interposed to prevent the approximation of the handles of the bellows, and the air is consequently expelled in part only, the range of the blowing capacity in the bellows is evidently diminished just in proportion as the expulsive movement is prevented. In an analogous manner the permanent expansion of the lungs in emphysema, by opposing an obstacle to the movements of expiration, restricts the extent of breathing capacity.

How does this compromise the respiratory functions? There is no deficiency of air within the cells of the lungs.— An over abundance of air, in fact, belongs to the morbid condition. But it is residual or stagnant air which is abundant. The volume of moving or tidal air, in the acts of inspiration and expiration, falls below the quantity required tor the function of respiration. The respiratory function is dependent on a sufficient quantity of moving air. This being inadequate, the blood fails to receive from the air inspired a sufficient quantity of oxygen, and the expired current fails to carry away from the blood a sufficient quantity of carbonic acid gas. Hence the interchange of these elements between the blood and the atmosphere is incomplete. Dyspnœa and lividity are incidental to the imperfect accomplishment of the respiratory function. The patient feels a painful sense of the want of fresh supplies of air. This constitutes the symptom called dyspnœa. Impelled by this suffering from the want of air, he instinctively employs laborious efforts to obtain it. Hence are called into play all the muscles which enlarge the capacity of the chest in the act of inspiration, and which compress the lungs by contraction of the chest in the act of expiration. But if emphysema exist to such an extent that the most laborious efforts of breathing are insufficient to satisfy the objects of respiration, not only does the dyspnœa continue, but the imperfection of the blood changes which should be wrought by the respiratory function, are manifested by lividity in parts where the vermilion hue of arterial blood is conspicuous in health, viz. The prolabia, the mucous membrane within the mouth, and, to some extent, the cutaneous surface, especially on the face.

There is another mode in which emphysema compromises the respiratory function, viz. By impeding the circulation of the blood through the lungs. I have pointed out the exsanguine appearance in the specimen on the table. For the same reason that the lungs are exsanguine after death, the blood is deficient in these organs during life. The air in the dilated cells compresses the capillary terminations of the pulmonic artery, and in this way obstructs the passage of blood through the pulmonic circuit. The function of respiration, therefore, suffers from the want of a sufficient supply of blood to the lungs, as well as from the want of a sufficient supply of fresh air. This obstruction to the circulation through the lungs necessarily lends to an accumulation of blood in the right cavities of the heart, and to stagnation in the systemic veins and capillaries. The lividity is partly attributable to this stagnation, as well as in part to deficient oxygenation of the blood. As a result of an accumulation of blood in the right cavities of the heart, they become distendered and enlarged, and ulterior effects which may occur are cardiac hypertrophy and dilatation.

If the anatomical condition which constitutes emphysema be understood, together with the mode in which it compromises respiration and interferes with the circulation, the symptomatology of the affection will be sufficiently intelligible. The signs obtained by the physical exploration of the chest are also understood without difficulty. I do not propose to enter into a consideration of these at this time. I shall consider them in connexion with other cases of emphysema which will come under our observation during the winter. I will simply say that you have only to keep before the mind's eye the permanent expansion of the lungs as you now see them, to understand that, when situated within the chest, the latter will be enlarged in proportion to the increased volume of the pulmonary organs; that the normal oblique direction of the ribs will be diminished in proportion as the thoracic walls are elevated and expanded, and the direction will approximate to a horizontal line; that the costal movements will be less than occurs in labored breathing in health; that, inasmuch as there is no deficiency of air in the air cells, the chest will be sonorous on percussions, and that, since the range of movements of the lungs and chest is diminished, the respiratory sound obtained by auscultation will be weakened. There are certain modifications of the form of the chest, of its motions, of the percussions note, and of the rhythm of the respiratory murmur, which constitute positive physical signs of this affection.— These, I shall point out to you on other occasions.

I will now give from my Hospital Book a condensed account of the previous history of the case which has served as a text for this lecture, together with the symptoms and physical signs. I shall not stop to offer any comments but I shall afterwards devote the remainder of the lecture to some remarks on the pathology and treatment of emphysema.

The patient, a laborer, aged 36, was admitted into the hospital fifteen days before his death. He had been in the hospital eleven days when my time of service commenced. He stated that he was well and strong up to two years ago. He then began to notice deficiency of breath on active exercise. About the same time (he was not certain whether shortly before or after) he began to cough and expectorate. The deficiency of breath, cough, and expectoration progressively increased, but he kept at work until August last, when he was obliged to give up. He did not take the bed till he entered the hospital. When he entered he was greatly prostrated. The lower extremities were œdematous. The dyspnœa was urgent. He was treated, before he came under charge, with the syrup of morphia, brandy, good diet, and occassionally the brown mixture. His condition became, in some respects, improved. The œdema of the lower extremities disappeared. He seemed less prostrated.

When I took charge of the patient, the cough was frequent and spasmodic, and he expectorated abundantly muco-purulent matter. The breathing was greatly labored, the inspirations spasmodic, the respirations numbering 36. The face and lips were tumid. The prolabia and tongue were livid, and the face also presented a livid hue. The appetite was good and the bowels regular. The pulse was 120. The surface was cool. The dyspnœa did not prevent him from lying down, and he preferred to lie on the left side.

On physical examination of the chest, the ribs and sternum were raised in inspiration, as if they formed one piece. The larynx descended in each inspiration. The chest presented at its upper part a barrel shape. The lower part was contracted in inspiration. The obliquity of the ribs was diminished. The percussion sound was everywhere clear. The resonance extended over the præcordia. The sonorousness was not great, but the chest was pretty thickly covered with muscle. The respiratory murmur in front, on the left side, was extremely weak. The inspiratory sound was notably deferred. On the right side, the respiratory murmur was less weak, the expiratory sound prolonged and lower in pitch than the inspiratory. Behind, the murmur was scarcely perceived on the left side, and was feeble on the right side. Sibilant rales were frequent on both sides, and, here and there, mucous rales. The apex-beat of the heart was not discoverable. An obscure impulse was felt in the epigastrium just below and to the left of the ensiform cartilage. The heart sounds were scarcely audible in the præcordia, either at the situation of the base or apex. Both sounds were heard in the epigastrium, the second sound being louder than the first. No cardiac murmur was discovered.

The urine was not albuminous, and I may state in this connection, that the kidneys after death presented a normal appearance.

My treatment consisted of the chlorate of potassa, half an ounce daily, the syrup of morphia *pro re nata*, brandy three onnces three times daily, and good diet.

The labor of breathing and the suffering from the want of breath continued. The lividity increased, and death took place on the fourth day after the patient came under my charge.—*Am. Med. Timss.*

